# Left Colon Diverticulitis Presenting as Perforated Lumbar Abscess: A Case Report and Review of the Current Literature

**DOI:** 10.1155/2015/414905

**Published:** 2015-12-31

**Authors:** Daniel Paramythiotis, Konstantinia Kofina, Vassileios N. Papadopoulos, Antonios Michalopoulos

**Affiliations:** 1st Propedeutic Surgical Clinic, Aristotle's University of Thessaloniki, AHEPA University Hospital, Stilponos Kyriakidi 1, 54636 Thessaloniki, Greece

## Abstract

Diverticular perforation is a common complication of diverticulitis and can lead to the creation of abscesses. The presence of such abscesses on the abdominal wall is rare and can lead to misdiagnosis. We present the case of a patient with abdominal pain and the formation of a large left lumbar abscess due to perforation of a diverticulum of the left colon and our surgical treatment of choice with favorable results.

## 1. Introduction

Diverticular disease affects mainly patients over forty years of age especially in the western countries, with left colon representing the most common site of presentation [1]. Most patients remain asymptomatic, while 20% of cases present symptoms [2]. Risk factors include obesity, smoking, and low-fiber diet, but the pathophysiology may also include a connection between diverticulitis, inflammatory disease, and irritable bowel syndrome [3]. Possible complications include abscesses, perforation, strictures, or the formation of fistula, altogether of a rate of 25% [2].

Actually, diverticulum perforation may, usually, lead to abscess formation because of the inflammation of the nearby structures through tissue continuity. However, the presence of an abscess on the abdominal wall due to such perforation is rare. We present the rare case of a patient with a large perforated left lumbar abscess and vague abdominal pain that was diagnosed as diverticular perforation and was treated surgically, following the existing guidelines [4].

## 2. Case Report

A 50-year-old male patient was admitted at the Emergency Department of our clinic complaining about mild diffuse abdominal pain, with no specific location of tenderness. This pain presented the previous day, along with fever up to 38.3°C. He did not mention any other symptoms, such as nausea, appetite loss, or bowel dysfunction. Prior to our examination, the patient was referred to the Internal Medicine Emergency Department, where he was scheduled to have a blood test and an X-ray examination of the abdomen (Figure 2). His laboratory values included WBC 8.43 K/*μ*L with 74.5% neutrophils, Hb 11.2 gr/dL, and Ht 34.3%. His vital signs were between normal ranges. From his medical history he revealed two episodes of acute diverticulitis during the past five years (five and three years before this admission, each), for which he followed conservative treatment. At the time of admission, he was not on immunosuppressant or any other medication.

**Figure 1:**
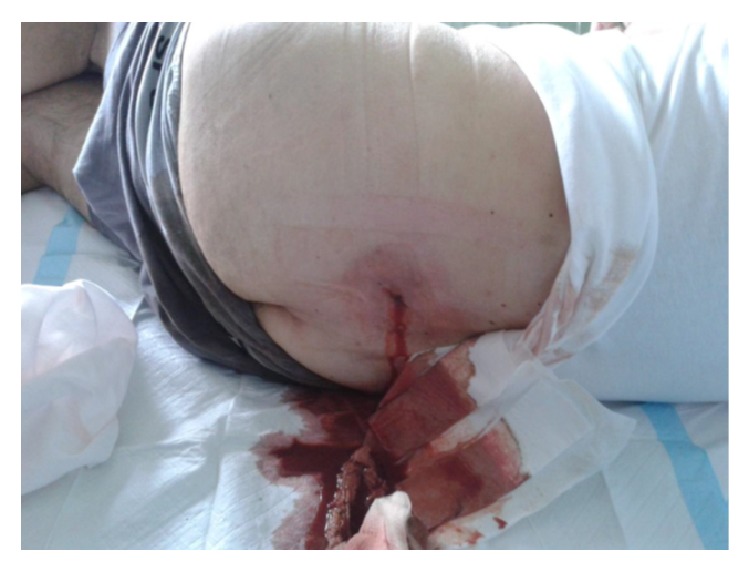
Image of left lumbar abscess drainage due to perforated diverticulitis.

**Figure 2:**
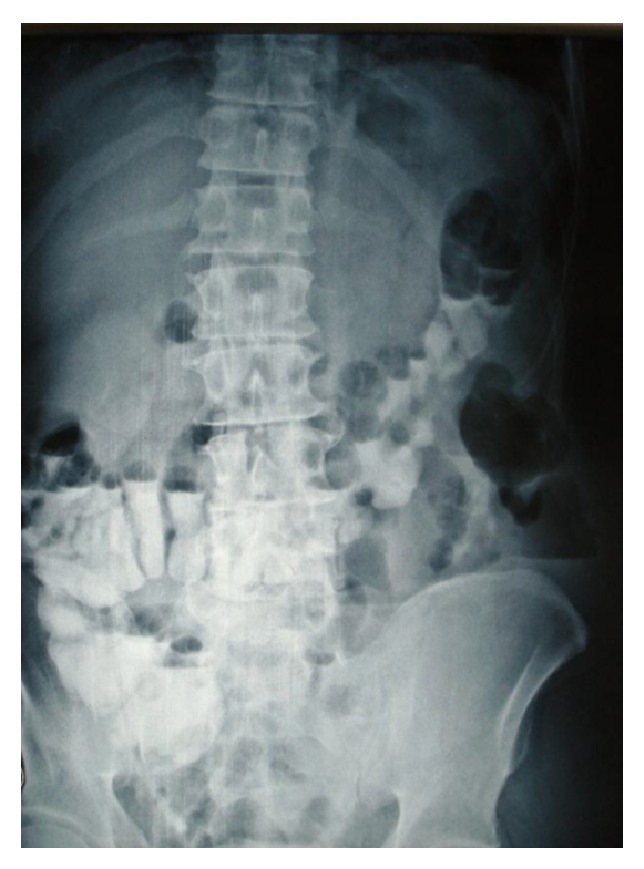
Abdominal X-ray showing inflammation at the left lumbar area.

**Figure 3:**
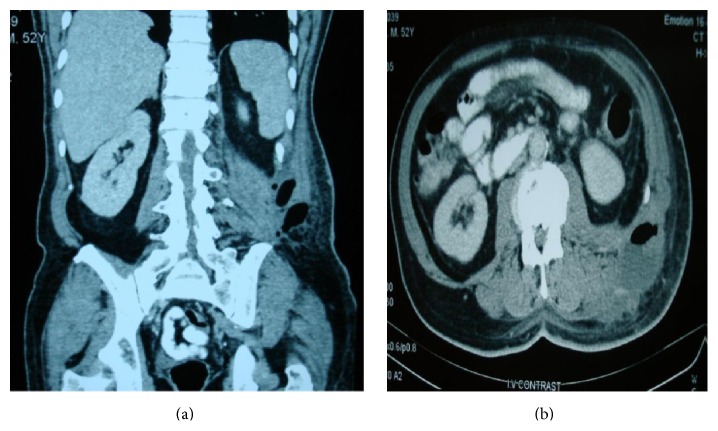
Abdominal CT scans revealed a perforated diverticulitis of the sigmoid colon causing subcutaneous inflammation and free gas in the tissue of the posterior abdominal wall and lumbar area.

During the clinical examination, a mild diffuse sensitivity during palpation of the left abdominal area was observed, without any specific signs of abdominal pain. However, the patient's inspection revealed an abscess in the left lumbar area, which, according to the patient, was present for about three months and tended to reduce in size after defecation. The abscess seemed uncomplicated, not too large, and manageable through drainage under local anesthesia on bedside. Therefore, it was incised, but almost two liters of pus-like fluid was drained (Figure 1). Even though there was no clinical suspicion for the performance of an abdominal CT prior to the drainage, the quantity and quality of the drained fluids made it necessary to carry out this examination for further control. The CT revealed the presence of free air in the lumbar wall, in the abscess area (but not in the abdomen), as well as signs of inflammation (Figures 3(a) and 3(b)).

**Figure 4:**
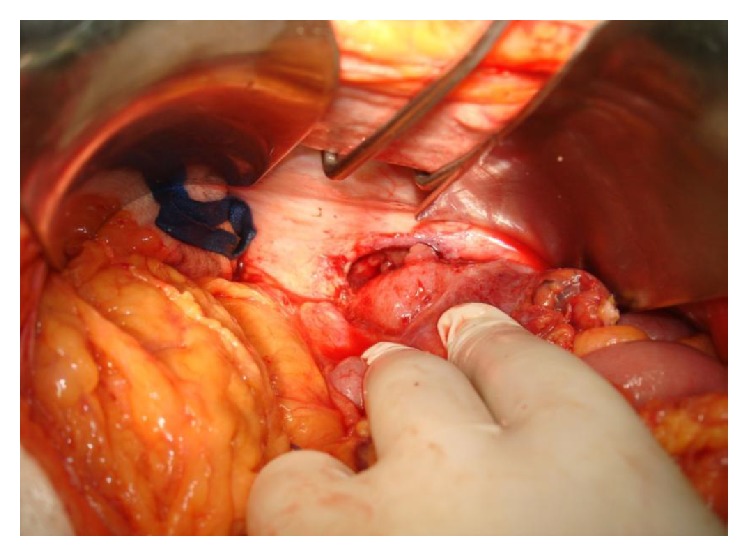
Intraoperative image of the loft colon perforation.

**Figure 5:**
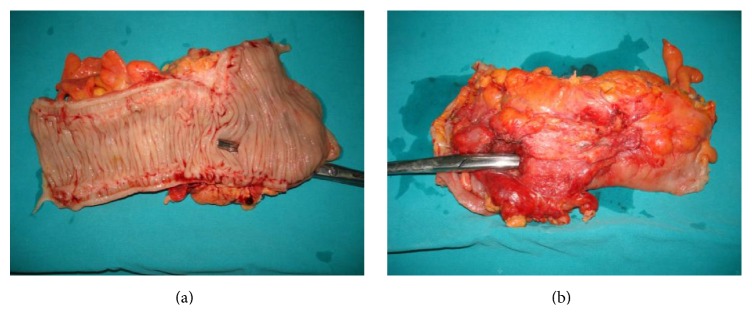
Postoperative excised left colon specimen.

Due to these imaging findings in combination with the patient's condition, we decided to perform an exploratory laparotomy, during which a perforated diverticulum of the left colon was revealed. This perforation led to the inflammation of the posterior abdominal wall through tissue continuity and resulted in the formation of the lumbar abscess. However, there were no signs of generalized peritonitis, as the fascia was not infected. The inflamed segment of the colon was excised at healthy margins and an end-to-end anastomosis was performed (Figures 4, 5(a), and 5(b)). The postoperative period was uneventful and the patient was discharged on the seventh day after surgery with the wall trauma on a good condition and with a recommendation of antibiotic uptake for the following two weeks.

## 3. Discussion

Diverticulitis is staged according to the classic Hinchey classification as pericolic abscess or phlegmon (Hinchey I), pelvic, abdominal, or retroperitoneal abscess (Hinchey II), generalized purulent peritonitis (Hinchey III), and generalized fecal peritonitis (Hinchey IV). Many other classifications have been reported also (Hughes, modified Hinchey, Köhler, Hansen/Stock, and Siewert) [5]. The usual presentation of a diverticulum perforation is acute abdominal pain and peritonitis. However, it can also appear with nontypical signs, through complications such as chronic abscesses, the creation of fistulae, and bowel obstruction. Therefore, cases have been reported that presented with colovesical and colovaginal fistulae [6], perianal fistulae [7], abscess formation on the abdominal wall [1], the thigh [6], or the renal fossa during end-stage renal disease [8], subcutaneous emphysema [9], and a perforation in a lumbar hernia [10].

In our case, the perforation appeared as a left lumbar area abscess of three-month duration, for which the patient did not seek treatment before, as he did not present any other symptoms. Apart from our case, only two other cases have been described clearly as an abscess in the lumbar area following diverticulum perforation in the literature (Table 1) [11, 12]. It is believed that recurrent attacks of diverticulitis provoke scarring and adhesion formation, so localized perforations and abscesses may appear instead of generalized peritonitis [4].

The management of acute diverticulitis includes fluid and antibiotic support, percutaneous drain abscess drainage or resection, and 1- or 2-stage anastomosis, depending on each individual case [13]. The first colon resection for the treatment of a diverticular perforation was reported by Mayo in 1907. New strategies in the management of perforated diverticular disease suggest that, in patients with Hinchey I and Hinchey II perforated diverticulitis, we should first perform laparoscopic lavage and secondly colonic resection and anastomosis at the same time. In Hinchey III and Hinchey IV perforated diverticulitis, the colorectal surgical specialists recommend either emergent definitive sigmoid resection with primary anastomosis with or without protective ileostomy formation or Hartmann's procedure [14].

Many studies have shown improved results through primary anastomosis, depending on the surgeon's experience [15], while others consider it as a frequent surgery with no higher morbidity or mortality in comparison to Hartmann's procedure [16]. In our case, resection of the damaged colon and primary end-to-end anastomosis were considered viable and the recovery of the patient was uneventful.

Abdominal CT is the imaging examination of choice, as it sets the definite diagnosis, shows the extent of abdominal inflammation, and excludes any other causes of acute abdominal pain [9]. In our case, a CT was immediately performed after the lumbar abscess drainage because, in combination with the abdominal pain, the X-ray findings, and the medical history, a clinical suspicion of diverticular perforation was set. The early diagnosis is crucial, as a delayed diagnosis of such perforation is associated with high mortality. As this presentation of diverticular perforation is not typical, a surgeon should be always aware of this possibility and prevent any misdiagnosis.

## 4. Conclusion

The diagnosis of a diverticular perforation, especially when it appears with nonspecific symptoms as a lumbar area abscess, may be difficult to establish and is often misinterpreted. A detailed clinical history must be taken and the patient should be examined thoroughly. Imaging findings such as CT can help in the differential diagnosis and lead to the precise diagnosis and to early surgical treatment.

## Figures and Tables

**Table 1:** Lumbar abscesses due to diverticular perforation: three reported cases and treatment of choice.

Authors	Year	Patients	Treatment
Green and Joypaul [11]	2009	1	Sigmoid colectomy and primary anastomosis

Coulier et al. [12]	2012	1	2-step surgical approach

Paramythiotis et al.	2015	1	Elective sigmoid dissection and primary anastomosis
